# Nystagmus characteristics and their impact on pattern-reversal VEP in patients with albinism

**DOI:** 10.1007/s10633-025-10026-1

**Published:** 2025-06-03

**Authors:** Herman E. Talsma, Gerard C. de Wit, Charlotte C. Kruijt, Stefan H. L. Zwerver, Maria M. van Genderen

**Affiliations:** 1 Bartiméus Diagnostic Center for Complex Visual Disorders, Van Renesselaan 30a, 3703 AJ Zeist, The Netherlands; 2 Department of Ophthalmology, Leiden University Medical Center, Leiden, The Netherlands; 3 Department of Cognitive Neuroscience, Donders Institute for Brain, Cognition and Behaviour, Radboudumc, Nijmegen, The Netherlands; 4 Department of Ophthalmology, University Medical Center Utrecht, Utrecht, The Netherlands

**Keywords:** Albinism, Nystagmus, Visual evoked potentials, Pattern-reversal VEP, Visual acuity, Eye movements

## Abstract

**Purpose:**

To describe the association between nystagmus characteristics and pattern-reversal VEP (prVEP) P100 amplitude and peak time in patients with albinism.

**Methods:**

We analyzed the prVEP (60’ and 15’ checksize) and nystagmus recordings from 47 patients with albinism, 17 with a relatively good visual acuity (≤ 0.3 logMAR), and 30 with a relatively poor visual acuity (≥ 0.6 logMAR). The nystagmus waveforms were classified into two types: dominantly pendular and dominantly jerk. We correlated the nystagmus type, amplitude, frequency, and percentage of low velocity (PLOV) to P100 amplitude and peak time.

**Results:**

For most patients (87%), reproducible responses were observed for the large checks (60'). Among patients with good visual acuity, 94% had reproducible responses of which the majority (82%) of P100 amplitudes fell within the normal reference range. In contrast, although 83% of patients with poor visual acuity showed reproducible responses, only a minority (17%) of P100 amplitudes were within the normal range. The P100 amplitude to 60’check sizes was statistically correlated with PLOV (r = 0.58, *p* < 0.0001), nystagmus type (r = −0.55, *p* < 0.0001), and nystagmus amplitude (r = −0.39, *p* = 0.0092).

Patients with relatively good visual acuity and jerk nystagmus exhibited the highest PLOV and the largest P100 amplitude (*p* < 0.0001). In contrast, there was no significant correlation between P100 peak time and any nystagmus parameters in patients with good or poor visual acuity.93% normal peak time. For the small checks (15’), 76% of patients with good visual acuity, still showed reproducible responses, with the majority (71%) of P100 amplitudes falling within the normal reference range. In contrast, among patients with poor visual acuity, only 3% (1 patient) showed reproducible responses, but with amplitudes below the normal range. For the patients with good visual acuity, PLOV showed a significant correlation with P100 amplitude. P100 peak time was normal for 77% (10/13) of these patients.

**Conclusions:**

For the prVEP with 60’ checks, nystagmus in patients with albinism predominantly affects the P100 amplitude but not the P100 peak time. For 15’ checks the amplitude is often so small that clear responses are no longer discernable, especially in patients with poor visual acuity.

**Supplementary Information:**

The online version contains supplementary material available at 10.1007/s10633-025-10026-1.

## Introduction

Nystagmus is a rapid, involuntary rhythmic movement of the eyes and is associated with various congenital conditions such as albinism, achromatopsia, congenital stationary night blindness (CSNB), and infantile idiopathic nystagmus (IIN). It can be described by four parameters: waveform, direction, frequency, and amplitude.

Congenital nystagmus generally leads to reduced visual acuity [1–10]. In the literature, several methods have been proposed to predict visual acuity based on the nystagmus parameters [1, 2, 4–7]. In these methods, the visual acuity is related to the so-called foveation periods [11], the period in the nystagmus cycle in which the eye velocity is low and the gaze position is close to the target.

Nystagmus not only affects a patient’s vision but also hinders testing the integrity of the optic nerve using visual evoked potentials (VEPs). Normally, the pattern-reversal VEP (prVEP) is the best choice for assessing optic nerve function since the peak time of the P100 component shows relatively little variation between subjects, between eyes, and over time [12, 13]. Unfortunately, especially the prVEP seems to be affected by nystagmus, much more than VEPs to other stimuli like pattern onset and flash [12]. Hoffmann et al. studied the effects of simulated horizontal nystagmus on the prVEP in normal subjects and concluded that the prVEP responses get attenuated because of the retinal image motion [14].

The purpose of this study was to investigate the effect of nystagmus characteristics on the prVEP in people with albinism. Since nystagmus and visual acuity are correlated [15], we investigated two groups of patients with albinism: one with a relatively good visual acuity and one with a relatively poor visual acuity.

## Methods

### Study population

We invited 51 patients from a larger group of 522 patients with albinism [16] for prVEP and recording of their nystagmus using an eye-tracker. The patients were selected based on having either relatively good visual acuity (logMAR ≤ 0.3, n = 21) or poor visual acuity (logMAR ≥ 0.6, n = 30). Four patients with relatively good visual acuity were excluded because either the VEP or the nystagmus could not be properly analyzed, leaving us with 47 patients for this study. Of the 47 patients, 16 had a genetically confirmed diagnosis of albinism (see supplementary table S1); the remaining patients had a clinical diagnosis of albinism.

The median age of the patients was 24.0 years (range, 12–69 years). 26 patients were female, and 21 patients were male. The study was approved by the Medical Ethics Committee of Leiden University Medical Center and adhered to the tenets of the Declaration of Helsinki. Informed consent was obtained from the subjects after explaining the nature and possible consequences of the study.

### VEP

VEPs were recorded according to the ISCEV standard [12]. We used an Espion E3 system (Diagnosys LLC, Cambridge, UK) at a sampling rate of 1 kHz and a bandpass filter of 0.625 to 100 Hz. The stimulus consisted of a checkerboard pattern (check size 60′ and 15’; 2.45 reversals per second) with a field size of 20° horizontally and 15° vertically. Stimulation was binocular. Each result was based on the average of 64 sweeps. Two results were measured for each stimulus, and if a recognizable consistent P100 response was observed in both, it was considered valid and reproducible. The final averaged response was based on two sets of 64 sweeps.

### *Nystagmus recording with eye-tracking* [15]

We used a Tobii T60 XL eye-tracker with a 24-inch TFT widescreen monitor (Tobii Inc., Stockholm, Sweden), which records the gaze direction of the eyes with a sample rate of 60 Hz without the need for a chinrest or other apparatus. The stimulus consisted of a black dot on a grey background with an average luminance of 50 cd/m2 presented for 25 s, with the patient sitting approximately 60 cm away from the display. The recordings took place in undisturbed conditions in an office room. Tobii Studio software 3.3.2. was used to collect the nystagmus data of the albinism patients, after an initial standard five-point calibration procedure.

### Nystagmus data analysis

After visually selecting a section with good-quality nystagmus data, we determined various nystagmus parameters: direction, average amplitude, average frequency, waveform, and a derived parameter—the percentage of low velocity (PLOV).

*Nystagmus direction*: The recorded data was visually examined to assess the direction of the nystagmus (horizontal, vertical, or a combination of both).

*Nystagmus amplitude and frequency*: The average amplitude and frequency were calculated based on an algorithm to find repeating peaks and troughs around a moving average using Python’s function scipy.signal.find_peaks [17]. Visually it was verified that the algorithm found the correct peaks and troughs.

*Nystagmus waveform*: We classified the nystagmus waveform of the patients into either predominant pendular or predominant jerk according to the method proposed by Theodorou [18]_._ The way we implemented the Theodorou method is described in Appendix A. This appendix also gives a comparison between Theodorou's waveform analysis and the (visual) 12-fold classification method of Dell’Osso [19].

**Fig. 1:**
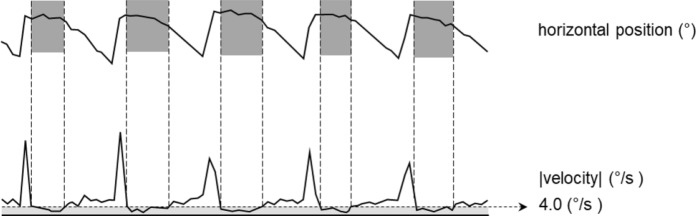
Example of the horizontal position of a jerk-type nystagmus (top graph) and the corresponding absolute value of the eye velocities (bottom graph). The top dark grey boxes indicate the slow phases. For this measurement interval, the percentage of low velocity (PLOV), defined as the proportion of time during which the velocity remains below 4°/s, is approximately 34%

*Percentage of low velocity (PLOV)*: earlier studies correlated visual acuity to the foveation periods of nystagmus [1, 2, 4–8, 10]. The foveation period is defined as the sections of the nystagmus cycle that have a low eye velocity in a certain gaze direction. In contrast to optotypes on a visual acuity chart, the stimulus of a pattern VEP is the same all over the screen. Therefore, we omitted the gaze direction criterium and defined the PLOV as the percentage of time during which the absolute velocity was < 4.0°/s [11]. Figure 1 illustrates the calculation of the PLOV. The top graph shows the horizontal gaze position as a function of time and the bottom graph the corresponding eye velocity. At the top also the periods in which the velocity is < 4.0°/s are shown. Over the whole period in the example, the eye had a velocity < 4.0°/s 34% of the time, resulting in a PLOV value of 34%.

### Statistical data analysis

All analyses were performed using GraphPad Prism version 6.0.7 for Windows (GraphPad Software, San Diego, California USA, www.graphpad.com). Since our study population is not an unbiased sample of all patients with albinism, we did not assume the VEP and nystagmus parameters to be normally distributed. For correlations between two parameters, we therefore applied the non-parametric Spearman test. We calculated the statistical differences between the group of patients with good and poor visual acuity with the Mann–Whitney U statistical test. A *p*-value of ≤ 0.05 was considered statistically significant.

## Results

### Nystagmus data

The direction of the nystagmus of all patients was horizontal (47/47). All other nystagmus parameters are given in Tables 1 and 2. Of the patients, 25 (53%) had a dominant jerk, and 22 (47%) had a dominant pendular nystagmus.

**Table 1:** Nystagmus data of 17 patients with albinism and good visual acuity: logMAR ≤ 0.3

Nystagmus parameter	PLOV (%)	Amp (°)	Frequency (Hz)
Min	2.3	0.2	1.5
5% percentile	3.3	0.7	1.5
25% percentile	11.3	1.8	2.2
50% percentile	18.7	3.0	2.7
75% percentile	34.8	7.4	4.0
95% percentile	44.0	12.1	5.2
Max	53.2	13.1	5.5

Nystagmus waveforms are given in  Table 3. PLOV = Percentage of low velocity

**Table 2:** Nystagmus data 30 patients with albinism and poor visual acuity: logMAR ≥ 0.6

Nystagmus parameter	PLOV (%)	Amp (°)	Frequency (Hz)
Min	2.8	2.2	1.7
5% percentile	3.4	3.1	2.0
25% percentile	5.2	4.4	2.7
50% percentile	7.7	6.4	3.1
75% percentile	9.7	7.6	3.7
95% percentile	16.3	11.3	4.1
Max	19.2	14.6	6.0

Nystagmus waveforms are given in Table 3. PLOV = Percentage of low velocity

**Fig. 2:**
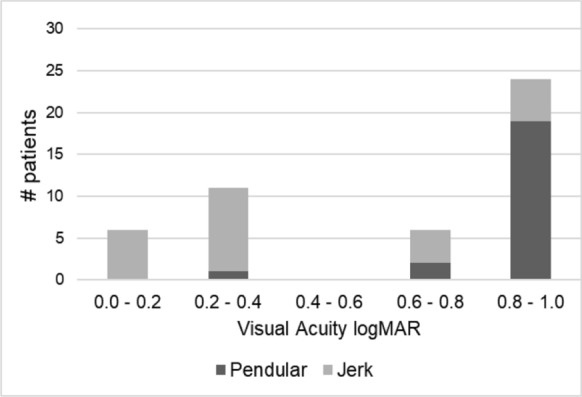
Histogram of the visual acuity (logMAR). Patients with pendular nystagmus had statistically significantly worse visual acuity (median 0.90 logMAR) than patients with jerk nystagmus (median 0.30 logMAR, *p* < 0.0001)

All but one patient with good visual acuity (0 ≤ logMAR ≤ 0.3) had jerk nystagmus (94%), with a median PLOV significantly higher (*p* = 0.0005) than those with poor visual acuity (0.6 ≤ logMAR ≤ 1.0), who predominantly had pendular nystagmus (70%), see also Fig. 4 (bottom left) and Fig. 2.

### prVEP P100 amplitude, peak time, and nystagmus parameters for large checks (60’)

**Fig. 3:**
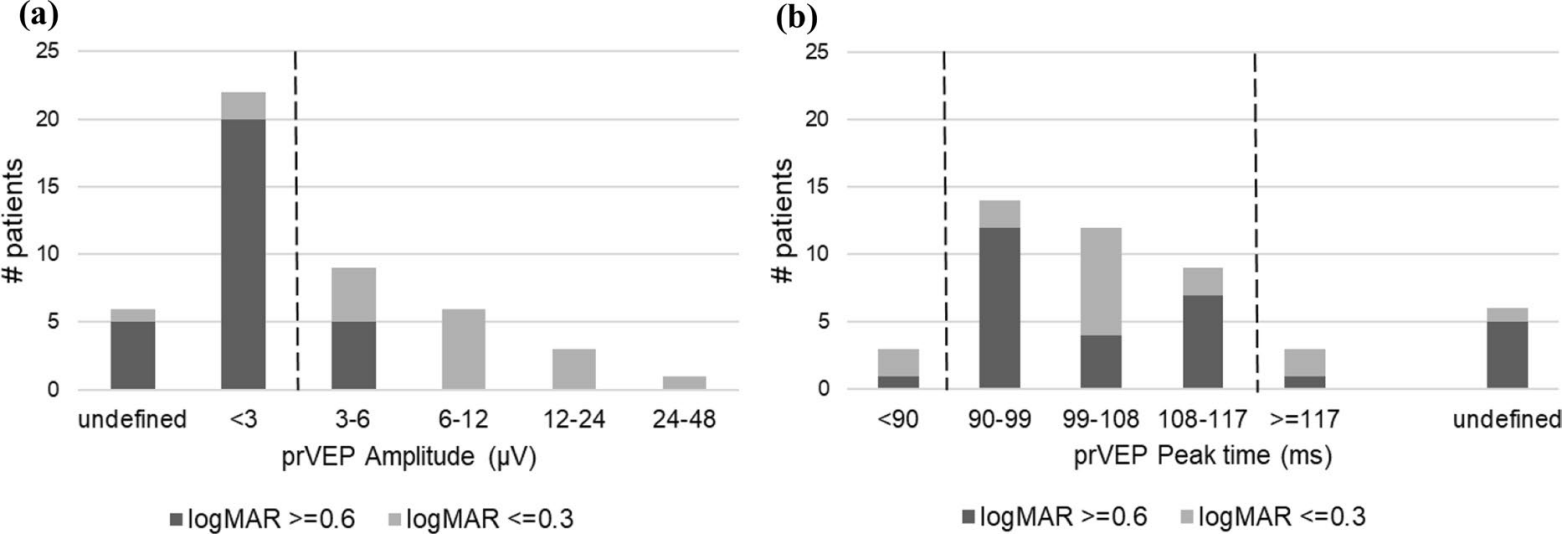
**a** Histogram of prVEP 60’ P100 amplitude, dashed line is the lower limit of the reference range. < 3 represents an amplitude smaller than 3uV but still recognizable. "Undefined" indicates an amplitude under 3uV, but so small that the P100 could not be recognized, and no peak time could be determined. **b** Histogram of the prVEP P100 60’ peak time, dashed lines are the lower and upper limits of the reference range

The P100 amplitude for 60’ checks ranged from 0 to 31 µV with a median amplitude of 2.7 µV. For patients with good visual acuity, P100 amplitude varied from 1 to 31 µV with a median amplitude of 6.8 µV and for patients with poor visual acuity, it ranged from 0 to 8.6 µV with a median amplitude of 2.3 µV. The observed disparity in P100 amplitudes between the group with good and poor visual acuity was statistically significant (*p* = 0.0001). In Fig. 3a it is evident that for most patients (87%), reproducible responses were observed. The majority of patients (94%) with good visual acuity had reproducible responses, and the majority (82%) of P100 amplitudes fell within the normal reference range. In contrast, for patients with poor visual acuity, 83% showed reproducible responses, while only a minority (17%) of P100 amplitudes were within the normal range. The P100 peak time ranged from 82 to 120 ms with a median peak time of 99 ms. For patients with good visual acuity, peak time varied from 84 to 120 ms with a median peak time of 100 ms. Patients with poor visual acuity had a peak time between 82 and 118 ms with a median peak time of 97 ms. There was no significant difference in P100 peak time between the group with good and poor visual acuity (*p* = 0.8).

As shown in Fig. 3b, 87% of patients exhibited a discernible peak. Of these, three patients (7%) had a peak time exceeding the upper reference limit. In six patients (13%), a peak time could not be determined due to an unrecognizable response.

**Fig. 4:**
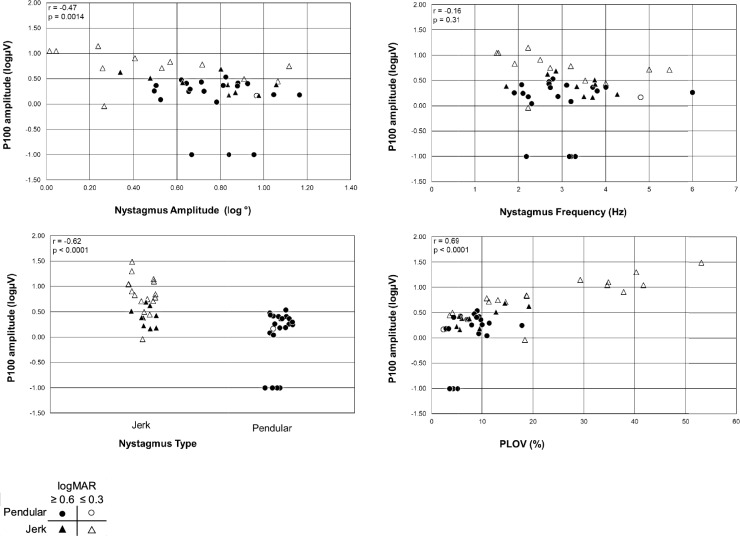
prVEP 60’ P100 amplitude as a function of nystagmus parameters. All nystagmus parameters except frequency correlated significantly. Triangles represent jerk nystagmus, and circles represent pendular nystagmus. Closed symbols indicate poor visual acuity (logMAR ≥ 0.6), while open symbols indicate relatively good visual acuity (logMAR ≤ 0.3). The P100 amplitude correlated most with PLOV and nystagmus type (see supplemental table S2). Data points at the log value of -1, represent a VEP amplitude of approximately 0 V

Figure 4 illustrates that, for the combined visual acuity cohort, the nystagmus parameters amplitude, type, and PLOV —but not frequency— correlated statistically significantly with the P100 amplitude. The parameters ranked by absolute correlation with the P100 amplitude from highest to lowest were as follows: PLOV (r = 0.69, *p* < 0.0001), nystagmus type (r = −0.62, *p* < 0.0001), log nystagmus amplitude (r = −0.47, *p* = 0.0014), and frequency (r = −0.16, *p* = 0.31). Even though no correlation with frequency was found for the combined visual acuity cohort, when analyzed separately, a significant correlation with frequency was identified in the good visual acuity cohort (r = −0.63, *p* = 0.025).

**Fig. 5:**
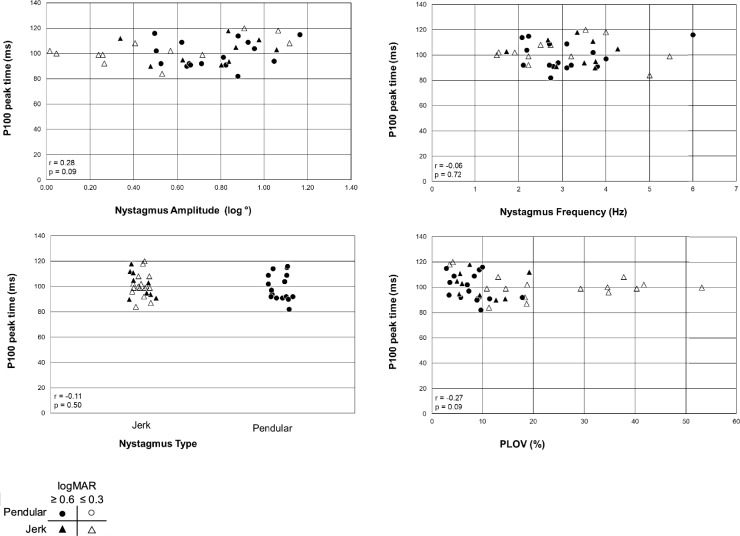
P100 peak time as a function of nystagmus parameters. None of the nystagmus parameters correlated statistically significantly with the VEP peak time. Triangles represent jerk nystagmus, and circles represent pendular nystagmus. Closed symbols indicate poor visual acuity (logMAR ≥ 0.6), while open symbols indicate relatively good visual acuity (logMAR ≤ 0.3)

None of the nystagmus parameters showed a significant correlation with the P100 peak time (Fig. 5). After splitting the cohort into patients with good and poor visual acuity, also no correlation was found (*p* > 0.05).

### prVEP P100 amplitude, peak time, and nystagmus parameters for small checks (15’)

**Fig. 6:**
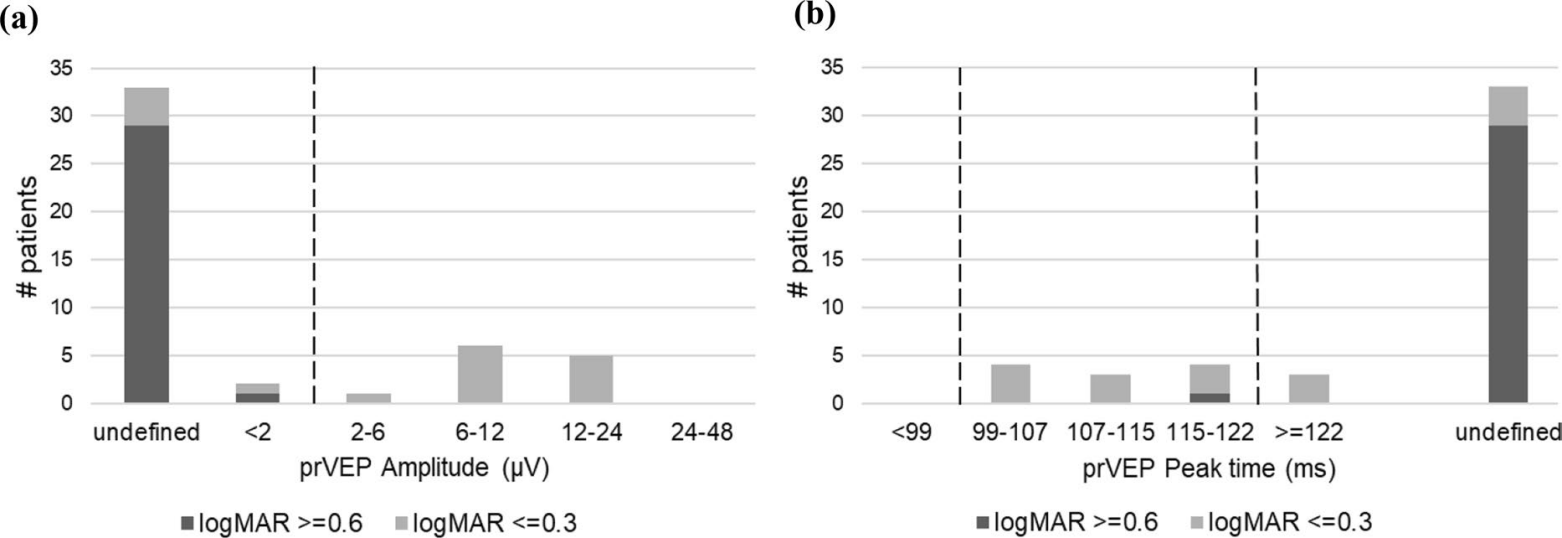
**a** Histogram of prVEP 15’ P100 amplitude, the dashed line indicates the lower limit of the reference range. **b** Histogram of the prVEP P100 15’ peak time, the dashed lines indicate the lower and upper limits of the reference range

The analysis of prVEP responses to 15' checks revealed markedly different results compared to the 60' checks. As illustrated in Figs. 6a and 6b, only one patient with poor visual acuity (3%) exhibited reproducible P100 responses. These responses were smaller than our reference limit. In contrast, most patients with good visual acuity (13/17 = 76%) showed reproducible responses, with only one patient having an amplitude below the normal reference range. Among patients with reproducible responses, a significant correlation was again observed between PLOV and P100 amplitude (r = 0.59, *p* = 0.029).

As shown in Fig. 6b, 21% of the patients with a reproducible P100 (3/14) had a peak time exceeding the upper reference limit. In the majority of patients (70%), no peak time could be determined due to a (nearly) absent response.

## Discussion

This study explored how nystagmus affects the prVEP P100 in patients with albinism. The majority of patients exhibited measurable prVEP responses. However, the reduction in amplitude observed in patients with poor visual acuity further emphasizes the influence of nystagmus on prVEP parameters.Strong correlations were identified between nystagmus parameters (PLOV, amplitude, type) and VEP amplitude, particularly in patients with good visual acuity.

Poor visual acuity was predominantly associated with pendular nystagmus, with only one exception in our cohort. Our findings align with previous studies [15, 16], showing that patients with jerk nystagmus tend to have better visual acuity than those with pendular nystagmus. The average nystagmus frequency (3.1 Hz) in our study was consistent with the findings of’Kumar et al. [20]findings (2.8 Hz), but the average nystagmus amplitude in our cohort (5.2°) was higher, likely reflecting the larger proportion of patients with poor visual acuity. The average amplitude of patients with good visual acuity (3.0°) closely matched the data of Kumar et al.

In contrast to half of the patients having a P100 amplitude below the reference limit, only 7% had a peak time exceeding the upper reference limit. Also, no significant correlation was found between nystagmus parameters and P100 peak time. For large checks (60’), the reduction in VEP amplitude, rather than a change in peak time, may be attributed to retinal image motion blur caused by nystagmus. Tumas et al. [21] observed a similar attenuation in prVEP amplitude with large checks (56’), attributing it to image blur, but smaller checks (14’) also led to prolonged peak times.

In our study, small checks (15’) yielded reproducible responses only in patients with jerk nystagmus and good visual acuity, while patients with pendular nystagmus and poor visual acuity failed to produce consistent responses. The combination of nystagmus and reduced visual acuity disproportionately impacted small checks, possibly explaining the prolonged peak times observed in some patients. Hoffmann et al. also examined the impact of simulated horizontal nystagmus on prVEP in normal subjects, attributing the attenuation of the signals to motion adaptation and image blur [14]. Our findings align with this, showing a strong correlation between PLOV and VEP P100 amplitude for the large checks (60’). For small checks (15’), the P100 amplitude in patients with poor visual acuity and nystagmus becomes so small that clear responses are no longer discernible. However, in patients with jerk nystagmus and relatively good visual acuity, the P100 amplitude remains detectable. For these smaller checks, we more frequently observed a prolonged P100 peak time compared to large checks, though the number of patients and therefore statistical power was smaller. Small checks are significantly more sensitive to factors affecting image quality, potentially resulting in prolonged implicit times, whereas larger checks remain unaffected [22–24]. In our study, the combination of nystagmus and foveal hypoplasia caused blur of the retinal image, disproportionately impacting the smaller checks and leading to the observed peak time prolongation in at least some patients. Both the type of nystagmus and the severity of foveal hypoplasia appear to influence visual acuity [15, 19] and prVEP, but the exact causal relationships remain unclear. Severe foveal hypoplasia is associated with pendular nystagmus and reduced visual acuity, but it is also linked to lower VEP amplitudes. However, it is difficult to determine whether foveal hypoplasia directly affects VEP responses or whether the presence of nystagmus plays a mediating role. The interplay between these factors suggests a complex relationship, where each parameter may contribute to the observed electrophysiological and functional outcomes, but their exact causal connections remain to be elucidated.A limitation of our study is that nystagmus complicates calibration during eye tracker measurements. While calibration was verified, accuracy may have been affected, though precision is less likely to be influenced. Since we focused on calculating amplitudes and correlating them with other parameters rather than examining gaze position, the lack of exact gaze position values is unlikely to have impacted our conclusions. Using only relative values in our analysis minimizes the potential effect of accuracy issues [25]. Another limitation of our study was the absence of real-time nystagmus recordings during prVEP measurements. The smooth gray background used for nystagmus recordings may have influenced nystagmus characteristics, though other factors, such as the contrast of the stimulus or the dynamics of visual motion, might also have contributed to the observed differences. Despite this limitation, our results show a clear correlation between nystagmus parameters (PLOV, amplitude, type) and prVEP P100 amplitude and a relatively unaffected peak time. Accurately characterizing nystagmus before testing could improve the reliability of prVEP as a diagnostic tool for patients with albinism or other conditions associated with nystagmus. Knowing the characteristics of a patient’s nystagmus beforehand can help clinicians anticipate whether obtaining a measurable prVEP response is likely. Alternatively, if an eye-tracker is used to identify moments when the eye is relatively stable, the reversals in the prVEP recording could be administered in these periods, ensuring more accurate measurements by minimizing the effects of retinal image instability. This opens up opportunities for the development of enhanced testing methodologies, such as integrating real-time eye-tracking technology to optimize stimulus timing. Efforts in this regard have already been undertaken by Talsma et al. [26] and Dunn et al. [27].

Our findings suggest that prVEP abnormalities may provide indirect clues about nystagmus type, particularly in clinical settings where eye-tracking is unavailable. Patients with pendular nystagmus and poor visual acuity often exhibited severely reduced P100 amplitudes, whereas patients with jerk nystagmus and relatively better visual acuity retained measurable responses, especially for larger check sizes. While this could be useful for clinical evaluation, particularly in young children where direct nystagmus assessment may be challenging, the relationship between prVEP findings, visual acuity, and nystagmus type remains uncertain. Further studies, including real-time nystagmus recordings during prVEP, are needed to clarify the potential role of prVEP in predicting nystagmus characteristics and visual function.

In conclusion, for 60’ checks, nystagmus in patients with albinism predominantly affects prVEP P100 amplitude and not the peak time. The amplitude decreases with reduced foveation time (smaller PLOV) and increased nystagmus amplitude, both of which are associated with poor visual acuity and pendular nystagmus. For 15’ checks, only some patients with a relatively good visual acuity (and jerk nystagmus) still give a discernable prVEP response.

## Electronic supplementary material

Below is the link to the electronic supplementary material.

Supplementary file1 (DOCX 38 KB)

## Figures and Tables

**Fig. 7:**
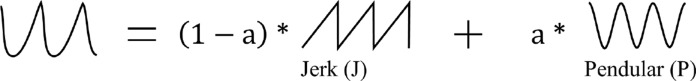
Nystagmus waveforms decomposition [18]

## Appendix A: Classifying nystagmus data as either predominantly jerk or pendular

Using Theodorou’s method [18], we decomposed the nystagmus into two waveform classes: dominantly pendular and dominantly jerk. This was done with the following procedure: First, with a Python program, we identified the different cycles of the measured nystagmus and for each cycle, the peaks and troughs were superposed by scaling the time axis. Subsequently, we discarded the outliers -cycles with log amplitudes that fell outside two times the standard deviation- and constructed an averaged and normalized waveform cycle. We then used Python’s scipy.optimize.curve_fit [28], non-linear least squares to best fit parameters a and w in the following function f, which is also visualized in Fig. 7: 

1 $${\text{f}} = \left( {1 - {\text{a}}} \right){\text{sawtooth}}\left( {2\pi t,1 - {\text{w}}} \right) + {\text{a}}(\cos 2\pi {\text{t}})$$

with parameter t the time, the sawtooth function representing the jerk term, the cosine function representing the pendular term, the factor a the amplitude of the pendular term, the factor 1-a the amplitude of the jerk term, and w the width of the rising ramp of the sawtooth function [29]. Parameter a falls for the normalized waveforms in the range 0–1. The width w of the rising ramp is a proportion of the total cycle. The default is 1, producing a rising ramp, while 0 produces a falling ramp. A width of 0.5 produces a triangle wave. Only the two extreme ranges for w were considered in the fitting: between 0.0 and 0.1, representing a right jerk, and between 0.9 and 1.0, representing a left jerk. If after fitting parameter a ≥ 0.5, then the waveform is considered predominantly pendular and if a < 0.5, then the waveform is considered predominantly jerk. The only nystagmus that cannot be classified this way is the bidirectional jerk, which we classified manually.

Some patients classified as having jerk nystagmus according to Dell’Osso's classification system [19] were categorized as having pendular nystagmus according to the Theodorou method [18] (Table 3). Patients classified based on Dell’Osso's pseudo-cycloid waveform fell into both the pendular and jerk classes according to Theodorou's classification.

**Table 3:** Nystagmus waveforms as defined by Dell’Osso and Theodorou reveal that some of Dell’Osso’s jerk types transitioned into the pendular class upon decomposition using Theodorou's method

n	Dell'Osso Type		Waveform	Theodorou
5	Pendular	Pure		Pendular
	Pendular	Assymetric		Pendular
4	Pendular	With foveating saccades		Pendular
-	Jerk	Triangular		Pendular
8	Jerk	Pseudo Jerk		Pendular
		Pseudo pendular pure		
		Pseudo pendular + foveating saccades		
6	Jerk	Pseudo cycloid		Pendular
2	Jerk	Pseudo cycloid		Jerk
2	Jerk	Dual Jerk		Jerk
5	Jerk	Bidirectional Jerk		Jerk
7	Jerk	Extended Foveation		Jerk
8	Jerk	Pure		Jerk
