# Supplementary material for: Nystagmus characteristics and their impact on pattern-reversal VEP in patients with albinism
Source: Doc Ophthalmol. 2025 Jun 3;151(1):45–56. doi: 10.1007/s10633-025-10026-1 (PMC12334444; doi:10.1007/s10633-025-10026-1)
Supplement: Supplementary file 1 — Supplementary file1 (DOCX 38 KB) [file 10633_2025_10026_MOESM1_ESM.docx]

**Supplemental data**

*Table S1. Overview of Patients with albinism sorted in the order of visual acuity, and nystagmus type. For patients who did not undergo genetic testing (*), we established the clinical diagnosis using the set of diagnostic criteria proposed by Kruijt et al* [16]*. In these cases, 3 major criteria or 2 major with 2 minor criteria were present for the diagnosis of albinism.* *In the presence of a molecular diagnosis, 1 major criterion or 2 minor criteria were sufficient.. Major clinical criteria would be (1) foveal hypoplasia grade 2 or more, (2) misrouting, and (3) ocular hypopigmentation, either iris translucency or fundus hypopigmentation grade 2 or more. Minor criteria would be (1) nystagmus, (2) hypopigmentation of skin and hair, (3) grade 1 fundus hypopigmentation, and (4) foveal hypoplasia grade 1.*

*Table S2a. Correlation prVEP P100 amplitude with nystagmus parameters.*

*Table S2b. Correlation prVEP P100 amplitude with nystagmus parameters, the group with poor visual acuity.*

*Table S2c. Correlation prVEP P100 amplitude with nystagmus parameters, the group with good visual acuity.*

*Table S3. Correlation prVEP 15’ P100 amplitude with nystagmus parameters*
